# Cave Diplopoda of southern China with reference to millipede diversity in Southeast Asia

**DOI:** 10.3897/zookeys.510.8640

**Published:** 2015-06-30

**Authors:** Sergei I. Golovatch

**Affiliations:** 1 Institute for Problems of Ecology and Evolution, Russian Academy of Sciences, Leninsky pr. 33, Moscow 119071, Russia

**Keywords:** Millipede, fauna, richness, cavernicoly, China, Oriental realm

## Abstract

The diversity of Diplopoda in caves of southern China is remarkably high, often 5–6 species per cave, consisting mostly of local endemics and presumed troglobionts. These are evidently biased to just a few lineages, mainly members of the orders Chordeumatida and Callipodida, the families Cambalopsidae (Spirostreptida) and Haplodesmidae (Polydesmida) or the genera *Pacidesmus*, *Epanerchodus* and *Glenniea* (all Polydesmida, Polydesmidae), *Trichopeltis* (Polydesmida, Cryptodesmidae), *Dexmoxytes* (Polydesmida, Paradoxosomatidae) and *Hyleoglomeris* (Glomerida, Glomeridae). All these taxa, especially the Paradoxosomatidae and Cambalopsidae (usually amounting to about 60% and 10% of the total species diversity in the Oriental fauna, respectively), are moderately to highly speciose across Southeast Asia, being largely epigean. However, the epigean Diplopoda of southern China are yet badly understudied, since much of the collecting and taxonomic exploration efforts still focus on cavernicoles. The Oriental Region is the only biogeographic realm globally that harbours all 16 orders of Diplopoda, of which 14 have already been encountered in China and/or the immediately adjacent parts of Indochina. Thus, China may actually prove to support no less than 1,000 millipede species of various origins, mainly Oriental and Palaearctic.

## Introduction

The class Diplopoda, or millipedes, is among the largest of the terrestrial arthropod groups globally, with about 8,000 described species in nearly 1,900 genera, 147 families and 16 orders (Shear 2011). However, the diversity of the Diplopoda is sometimes estimated at up to 80,000 species (Hoffman 1980), currently between 15,000–20,000 using some modern statistics (Brewer et al. 2012). The earliest millipedes are known from the Silurian (early Palaeozoic) and show remarkable ordinal-level diversity, with six orders recorded so far since the Carboniferous (late Palaeozoic) (e.g. Shear and Edgecombe 2010). Being so ancient and diverse taxonomically, widespread (present on all continents except Antarctica), virtually fully terrestrial (even fossils show spiracles), poorly vagile (with highly limited dispersal capacities) and highly limited in compensatory ecological faculties (strongly restricted by a single limiting ecological factor even if the others are favourable), millipedes have long been considered as an exemplary group for biogeographical studies and reconstructions (e.g. Shelley and Golovatch 2011).

Diplopods are largely detritivores, only a few species can be considered omnivores, even fewer as carnivores. The environment that can be postulated as the most typical of the Diplopoda as a whole is temperate (especially deciduous), subtropical or tropical forest (in particular, humid ones). The most typical habitats are leaf litter, the litter/soil interface, the uppermost soil, and dead wood. Being mainly hygro- to mesophilous, millipedes tend to be absent from or only marginal in most of the extreme habitats such as tundra or desert (Golovatch and Kime 2009).

Several basic millipede morphotypes are known: polyxenoid (Polyxenida), glomeroid (Glomerida and Sphaerotheriida), juloid (virtually all Juliformia), polydesmoid (Polydesmida, some Chordeumatida) and platydesmoid (Colobognatha). Similarly, five life-forms, or ecomorphotypes, have been delimited in millipedes (Kime and Golovatch 2000, Golovatch and Kime 2009). Thus, stratobionts, restricted to litter and the uppermost soil, are dominant in the Diplopoda and represented by all five morphotypes. Pedobionts, or geobionts, mainly restricted to mineral soil and represented by the smaller juloid, glomeroid and polydesmoid morphotypes, usually show body miniaturization or elongation, the shortening of appendages, often also decoloration of the teguments and the loss of eyes. Troglobionts, likewise represented almost entirely by the juloid, glomeroid and polydesmoid morphotypes, usually demonstrate a drastic elongation of the extremities, depigmentation of the teguments, blindness, sometimes mouthpart modifications and often also “cave gigantism”. Under-bark xylobionts, or subcorticoles, are also represented by all five morphotypes, but tend to be either particularly flat-bodied (polydesmoids, platydesmoids) or miniature (polyxenoids, glomeroids), often also especially thin (juloids). Finally, epiphytobionts, again with all five morphotypes involved, seem to be characteristic of suspended soil in warm humid forests and are characterized by very small body sizes. The life-form of epiphytobionts is still too poorly delimited to be sure. Moreover, since life-forms of arboricoles (= dendrobionts), symbionts of ants or termites, deserticoles etc. are habitually even less conspicuous, none of them seems to warrant the recognition of a separate life-form.

**Figure 1.**
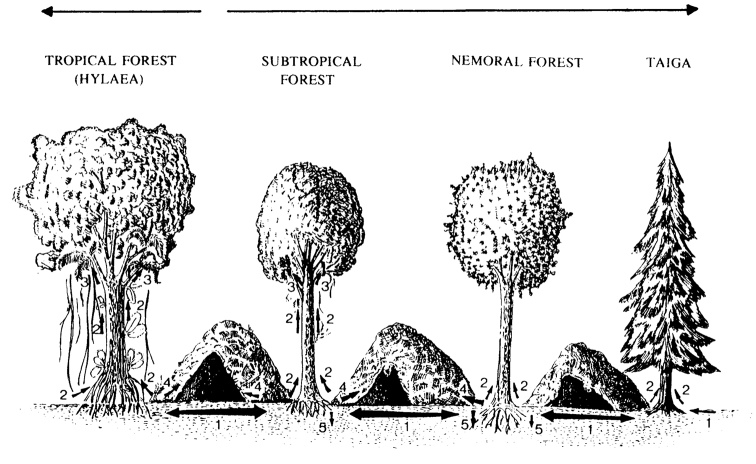
Main trends in the ecological evolution of Diplopoda. All are life forms except for arboricoles. **1** stratobionts **2** trunk and crown arboricoles, as well as subcorticolous xylobionts **3** epiphytobionts **4** troglobionts **5** geobionts. **NB**: The thickness of numbered arrows roughly corresponds to the share of the respective ecological grouping along a gradient of biome succession with age (uppermost arrows, the gap between them indicates the primary subtropical biome whence developed all the main extant biomes). After Kime and Golovatch (2000). Drawing courtesy S. Dashdamirov.

Figure 1 schematically depicts the main trends in, and pathways of, diplopod ecological evolution (Kime and Golovatch 2000). The biomes are arranged according to their age along two vectors of past climatic/biotic change, one showing deterioration from poor to worse conditions (overcooling from subtropical to boreal forest), and the other from warm to hot conditions (overheating, from subtropical to tropical forest) since the end of the so-called “warm Earth”, i.e. terminal Oligocene. Generally speaking, all derivative life-forms, i.e. subcorticoles, epiphyto-, troglo- and geobionts, may have evolved from the main stratobionts more or less simultaneously, relatively recently and perhaps since the onset of the Plio-Pleistocene glaciations. The few and rather indistinctly defined life-forms in Diplopoda seem to support this opinion. Yet one must distinguish the age of a taxon from that of a derivative life-form, because there are quite a number of relictual high-level diplopod taxa scattered across the world, including cavernicoles, whose origins seem to date back to the early Cenozoic or even Mesozoic times. We can assume that early in their evolution Diplopoda were generally detritivores living on the forest floor, and that this still applies to the majority. Cylindrical burrowers (juloids), flat-backed litter-splitters (polydesmoids) and rollers (glomeroids) are known already since the Palaeozoic. These were mainly large, spiny or crested forms, unequivocally stratobionts (Kime and Golovatch 2000).

To summarize, only a few millipede life-forms can be distinguished. Diplopoda as a group of soil/litter macrofauna are somewhat to markedly sensitive to water deficit and are often calciphilous as well. In general, they appear to have failed, both morphophysiologically and ecologically, to conquer environments and habitats significantly deviating from a forest floor (Kime and Golovatch 2000, Golovatch and Kime 2009).

## The fauna of China in the context of that of Southeast Asia

Holt et al. (2012), based on the modern distributions and phylogenies of amphibians, birds and non-marine mammals (altogether, 21,000+ species), have recently advanced a new terrestrial zoogeographical regionalization of the world, recognizing 20 major zoogeographical regions grouped into 11 larger realms. Among other novelties, a new, independent, Sino-Japanese region has been discriminated which also covers southern China and which appears to show closer phylogenetic affinities to the Palaearctic than to the Oriental realm. Eventually, that paper represents one of the most consistent, but no less unsuccessful attempts at uncritically combining the landscape-typological (= zonal) and faunogenetic approaches to biogeography which must be clearly separated at least as regards the biotas of older biomes (e.g. Chernov 1975). In addition, past distributions and the fossil record have been totally neglected, whereas some rivaling phylogenies preferred over others. As a result, based on the present-day borders of the tundra, steppe (= grassland) and desert zones, Holt et al. (2012) incorporated most of the tundra-clad northern North America into the Palaearctic and also distinguished a separate, arid, Saharo-Arabian region.

Heiser and Schmitt (2013), based on the distribution of the insect order Odonata in Eurasia and using various statistics, have recently provided an attempt at drawing a refined boundary between the Palaearctic and Oriental realms. The result was predictably clear and fully agreeing with common wisdom, as it shows a broad transition zone between these realms. Hardly surprisingly, this zone roughly corresponds to the Sino-Japanese region as delimited by Holt et al. (2012), naturally with southern China considered as its part.

Speaking of the Diplopoda, Southeast Asia is the only biogeographic region globally that supports all of its 16 orders (Shelley and Golovatch 2011). Because China together with the adjacent parts of Indochina alone encompasses at least 14 of these, its southern and central parts not only represent the northern periphery of the Oriental realm bordering on and intermingling with the Palaearctic one, but also a huge refuge harbouring numerous relict elements at various levels.

The following examples illustrate the declining millipede orders Siphoniulida and Siphonocryptida, neither reported from continental China yet.

All 2–3 species of Siphoniulida are only known from Sumatra (1) and Mexico+Guatemala (1–2). The pattern demonstrated by the Siphonocryptida is also quite peculiar. This small order contains two genera with three species in each. *Hirudicryptus* Enghoff & Golovatch, 1985 has a species living on Madeira and the Canaries, where it is largely confined to the relict laurisilva biome, one species from a 2500 m elevation in Nepal, and one from Taiwan. *Siphonocryptus* Pocock, 1894 contains one species from Sumatra, Indonesia, and two from the southern half of Malay Peninsula. Such patterns seem to date back at least to the Oligocene times of the so-called “Warm Earth” and have firm causal explanations (Golovatch 1997, Zherikhin 2003). Being so vastly disjunct, they are best accounted for by extinction events, extinction being as an integral part of evolution, both phylogenetic and spatial, as speciation. In contrast, most if not all of the remaining orders of Diplopoda are currently in an expansive stage of their evolution (Hoffman 1980, Shelley 2011).

## Troglobionts versus other life-forms

Faunistic records in southern China (not only of Diplopoda, but of many other arthropod groups) appear to be strongly biased towards caves; many if not most of the species are suspected or confirmed troglobionts, often with up to 5 or 6 troglomorphs per cave, for example in the Mulun Karst in Guangxi which hosts perhaps the richest cave fauna at least in China (Deharveng et al. 2008). We can easily predict that actually a far richer millipede diversity is represented by the other life-forms combined, both endogean, i.e. pedobionts, and above-ground, i.e. strato-, xylo-, epiphytobionts and, especially, stratobionts. Troglobionts, however common in the so cave-rich, karst-dominated, southern provinces of China, still constitute there only a subordinate fraction of the region’s overall diplopod diversity. This must never be forgotten when collecting anywhere, including China.

The cave millipedes of Southeast Asia, including the adjacent areas of southern China, in contrast to their epigean faunas, appear to be strongly biased and restricted to rather few lineages. In other words, even though the Oriental Region does support perhaps the richest and most diverse diplopod fauna globally (Table 1), the cavernicolous millipedes, however common in terms of abundance (quite often) and species richness, are represented by surprisingly few families and genera. Like elsewhere in the world, most/all of the troglobionts as a distinct life-form actually belong to the orders Spirostreptida, Glomerida, Chordeumatida, Callipodida and Polydesmida. The proportions of the few troglobitic species of Polyxenida, Glomeridesmida or Spirobolida known at the present are negligible (currently zero in the entire Oriental realm).

**Table 1.** Millipedes of Southeast Asia (and the world) versus those in southern China.

Orders	Distribution pattern	Troglobionts
Polyxenida	Cosmopolitan	very few troglobionts, but none in E & SE Asia
Glomeridesmida	Pantropical	very few troglobionts, but none in E & SE Asia
Glomerida	Holarctic + Oriental	numerous troglobionts, including E & SE Asia
Sphaerotheriida	Old World	no troglobionts
Siphoniulida	Neotropical + Oriental	no troglobionts
Siphonophorida	Pantropical	no troglobionts
Siphonocryptida	Palaearctic + Oriental	no troglobionts
Polyzoniida Platydesmida	Subcosmopolitan	no troglobionts
Chordeumatida	Holarctic + Neotropical + Oriental	no troglobionts
Callipodida	Subcosmopolitan, but mainly Holarctic	numerous troglobionts, including E & SE Asia
Stemmiulida	mainly Holarctic + Oriental	rather few troglophiles, but hardly any true troglobionts, which are mostly restricted to SE Asia
Julida	Pantropical	no troglobionts
	mainly Holarctic + Oriental	numerous troglobionts, including E Asia, but excluding SE Asia
Spirostreptida	Pantropical	numerous troglobionts, including E & SE Asia
Spirobolida	Pantropical	very few troglobionts, but none in E & SE Asia
Polydesmida	Cosmopolitan	numerous troglobionts, including E & SE Asia

Biogeographically, the millipede fauna of southern China, including cavernicoles, is clearly dominated by Oriental elements, whereas the influence of the Palaearctic is low. Such a pattern fully agrees with common wisdom. The same concerns the obvious preponderance of troglobitic Diplopoda to particularly local endemism, mostly restricted to a single cave or cave system, even as compared to the low-vagile and also mostly highly locally distributed epigean counterparts (Golovatch 1997).

## The main lineages of cave-dwelling Diplopoda in the Oriental Region and southern China

The most common, often also highly abundant group clearly dominating the cave millipede fauna of Southeast (and partly South) Asia is the family Cambalopsidae (Spirostreptida). The most speciose genera are *Glyphiulus* Gervais, 1847 (Golovatch et al. 2007a, 2007b, 2011b, 2011c, Golovatch et al. 2012b) and *Plusioglyphiulus* Silvestri, 1923 (Golovatch et al. 2009a, 2011a), followed by *Trachyjulus* Peters, 1864 (Golovatch et al. 2012a) and *Hypocambala* Silvestri, 1895 (Golovatch et al. 2011d). All of them largely contain epigean species, however at least two dozen *Glyphiulus* species (e.g. Fig. 10), plus one *Hypocambala*, are presumed troglobionts in southern China. The real diversity of Cambalopsidae in Chinese caves is difficult to estimate, but the number of likely troglobitic species of *Glyphiulus* alone may well amount to a hundred.

The huge, Eurasian, basically warm-temperate to tropical genus *Hyleoglomeris* Verhoeff, 1910 (Glomeridae, Glomerida) currently contains nearly a hundred species, including at least two dozen cavernicoles. Unlike the glomerid fauna of the adjacent Indochina which harbours a considerable proportion of endemic genera (60% in Vietnam, see Golovatch, Geoffroy and VandenSpiegel 2013), continental China currently supports only 23 species of *Hyleoglomeris*, of which over a dozen occur in caves alone (Golovatch, Geoffroy and VandenSpiegel 2012). According to our estimates, this figure may easily double or even triple with further studies on the cave Diplopoda of China.

**Figures 2–4.**
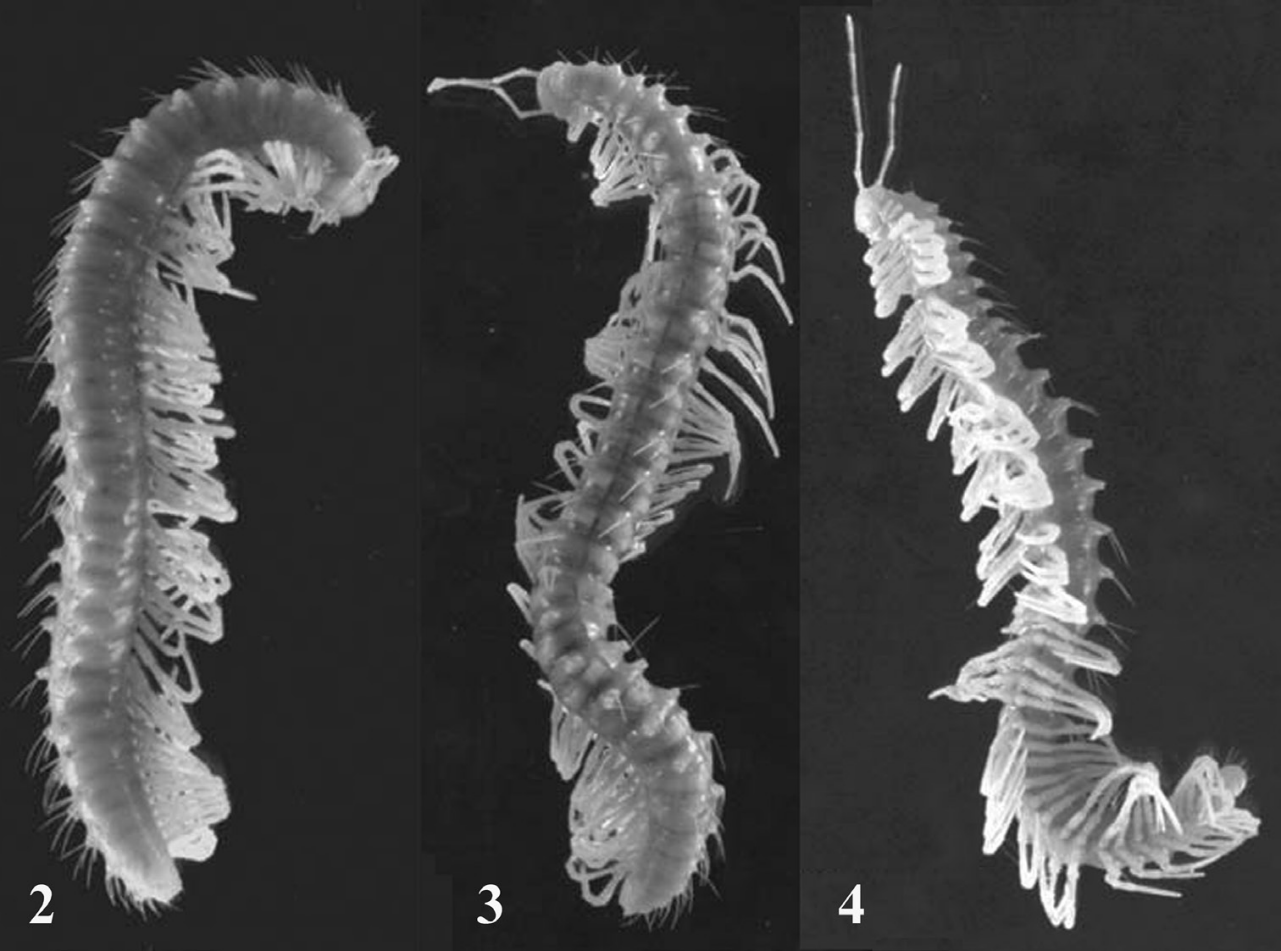
Habitus of *Nepalella grandoides* Golovatch, Geoffroy & Mauriès, 2006, a completely unpigmented, blind, long-legged and long-antenned troglobiont from Sichuan, length nearly 38 mm. After Golovatch et al. (2006b). Photos courtesy L. Albenga.

Species of the large order Chordeumatida dominate the Holarctic, being much more subordinate in Australasia (including southern India and Sri Lanka in the West, through Malay Peninsula and Indonesia, to tropical and subtropical eastern Australia and New Zealand), Madagascar (absent from the remaining Afrotropical areas), Central America (only north of Panama) and South America (only Chile). In Southeast Asia together with the adjacent areas of southern China, the fauna is restricted to a few genera only. The most important is *Nepalella* Shear, 1979 (Megalotylidae), with 23 species or subspecies from Nepal (10), Thailand (2), Myanmar (2), Vietnam (1) and southern China (8, several presumed troglobionts, e.g. Figs 2–4) (Golovatch et al. 2006a, 2006b). The oligotypic genera *Vieteuma* Golovatch, 1984 and *Lipseuma* Golovatch, Geoffroy & Mauriès, 2006 (both Kashmireumatidae), include one and two presumed troglobionts in southern China, respectively (Mauriès and Nguyen Duy-Jacquemin 1997, Golovatch et al. 2006a, 2006b). The small Oriental family Heterochordeumatidae (two genera and four species) is as yet unknown from southern China and contains no cavernicoles (Shear 2000). The same can be stated as regards the closely related, but much larger Metopidiotrichidae, with seven genera and over 50 species ranging from Japan, Taiwan, Indochina, Myanmar, Malaysia and the Philippines, through Indonesia, to Papua New Guinea, eastern Australia and New Zealand (Shear 2002). The only family-level endemic of southern China is the troglobitic monobasic genus *Guizhousoma* Mauriès, 2005 (Guizhousomatidae). Finally, also of relevance is the small family Pygmaeosomatidae which hosts only a few species in southern India, Sri Lanka and Madagascar, albeit none of them is cave-dwelling.

The rather small, mostly Holarctic order Callipodida is represented in the Oriental realm by 3–4 genera and three families. Perhaps the most interesting is *Sinocallipus* Zhang, 1993, with six described species, largely cavernicolous, from Indochina and the adjacent parts of southern China (Stoev and Enghoff 2011). This genus forms a distinct family and suborder, i.e. Sinocallipodidae and Sinocallipodidea, respectively, possibly the basalmost component in the entire order. Similarly, the genus *Paracortina* Wang & Zhang, 1993 (= ? *Angulifer* Zhang, 1997) is tropical or subtropical. At the moment it harbours 12 species, mostly cave-dwelling, in southern China and northern Vietnam, and it represents still another Oriental family of its own, Paracortinidae (review: Stoev and Geoffroy 2004). In contrast, the genus *Bollmania* Silvestri, 1896 (Caspiopetalidae) is chiefly Central Asian (south to Punjab in Pakistan). It contains only eight described species, mainly epigean, including a single troglobiont from Yunnan, southern China (Stoev and Enghoff 2005).

**Figures 5–8.**
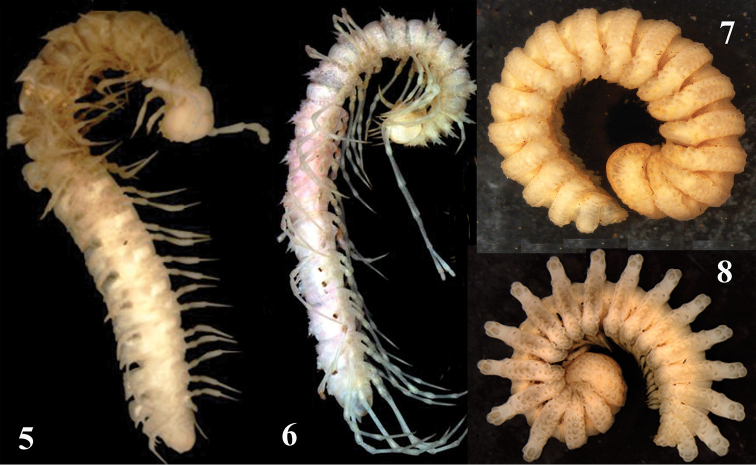
Habitus photos of *Pacidesmus armatus* Golovatch, Geoffroy & Mauriès, 2010, *Desmoxytes scolopendroides* Golovatch, Geoffroy & Mauriès, 2010, *Eutrichodesmus filisetiger* Golovatch, Geoffroy, Mauriès & VandenSpiegel, 2009, and *Eutrichodesmus aster* Golovatch, Geoffroy, Mauriès & VandenSpiegel, 2009, all presumed trogloionts from Guangxi, Guangxi, Vietnam, and Vietnam, respectively. After Golovatch et al. (2009c, 2010a, 2010b). Photos courtesy L. Deharveng & A. Bedos.

**Figure 9.**
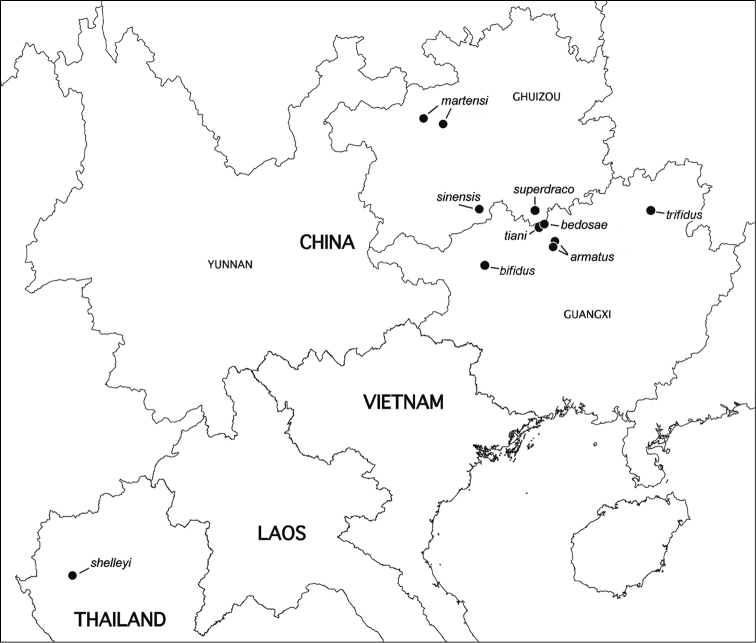
Distribution of *Pacidesmus* species. After Golovatch and Geoffroy (2014). Map courtesy L. Deharveng & A. Bedos.

Hardly surprisingly, the order Polydesmida, which is the largest globally, is also the most diverse in the Oriental Region. However, only a few families are represented in caves while even fewer seem to comprise troglobionts. The most common is the principally Holarctic family Polydesmidae only marginally represented in tropical Asia, reaching Indochina in the South. Two polydesmid genera dominate the fauna of China and adjacent areas, showing lots of cavernicoles as well. Thus, *Epanerchodus* Attems, 1901 is the largest genus of Polydesmida in Central to East Asia, including southern China. Altogether it contains 70+ species, mainly in Japan from where numerous troglobionts are known. Only 17 species of *Epanerchodus* have hitherto been recorded in mainland China (Golovatch 2014a, 2014b, Golovatch and Geoffroy 2014); at least 6 of them are presumed troglobionts which are all encountered only in southern China (Geoffroy and Golovatch 2004, Golovatch, Liu et al. 2012, Golovatch and Geoffroy 2014). The distribution of *Pacidesmus* Golovatch, 1991 (Figs 5 and 9) is even more spectacular: one species has been found at 2200–2500 m elevations in northern Thailand, whereas the remaining eight known congeners are troglobionts in southern China (Golovatch, Geoffroy and Mauriès 2010, Golovatch and Geoffroy 2014). Most of the few species of the basically Himalayan genus *Glenniea* Turk, 1945 are epigean, including one congener found in Guangxi (Golovatch, Liu et al. 2012); however, two species of *Glenniea*, one of which is fairly troglomorphic, have only been encountered in caves of Sichuan (Golovatch and Geoffroy 2014).

**Figure 10.**
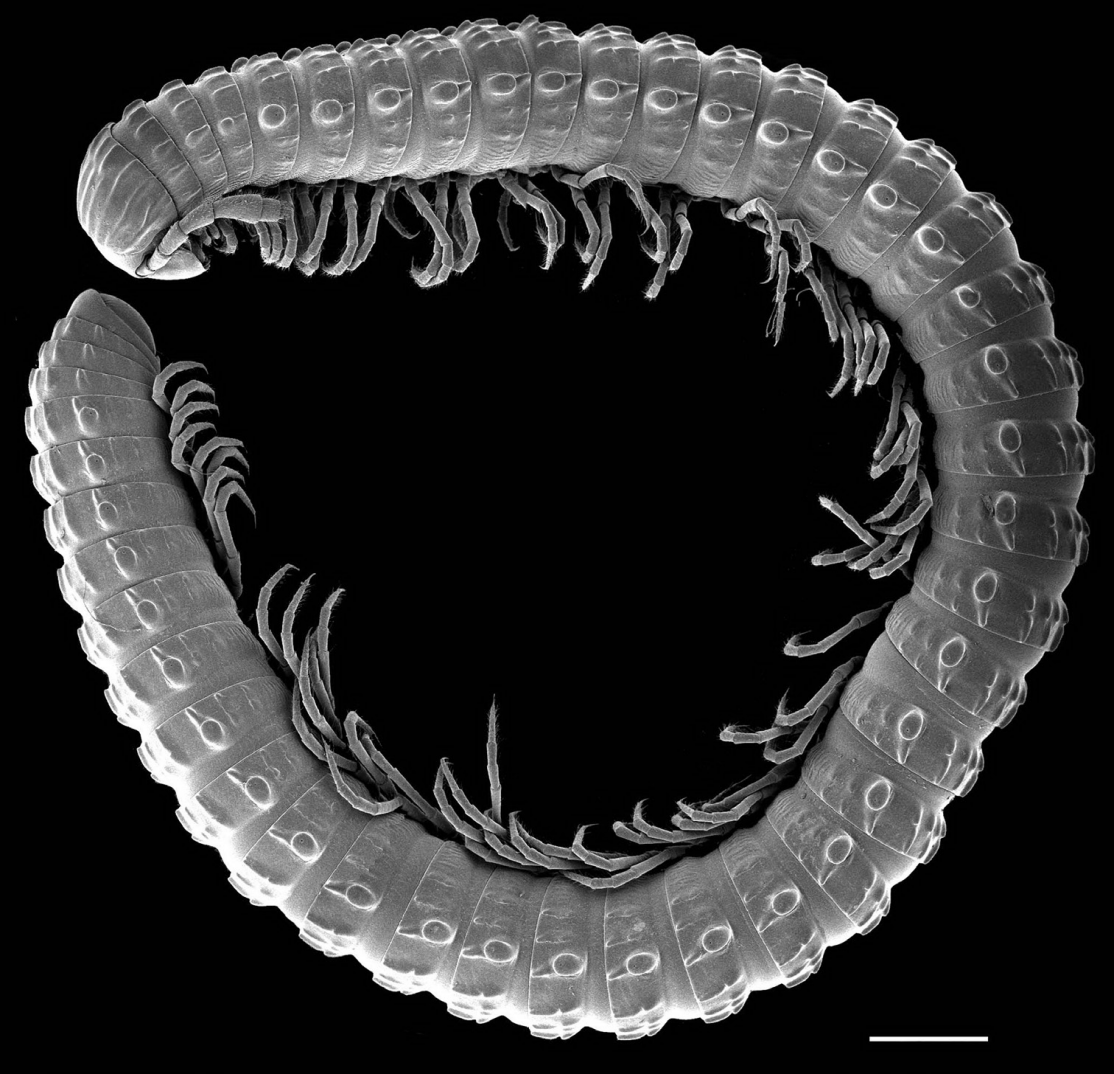
Habitus of *Glyphiulus difficilis* Golovatch, Geoffroy, Mauriès & VandenSpiegel, 2011, a presumed troglobiont from Guangxi. Scale bar: 1.5 mm. After Golovatch et al. (2011b). SEM micrograph courtesy D. VandenSpiegel.

The family Paradoxosomatidae (Polydesmida) is among the largest in the entire class Diplopoda (nearly 200 genera and >950 species, amounting to about 60% of the total species diversity in the Oriental fauna), but it is highly uncharacteristic of caves. Like the Cambalopsidae (probably another 10% of the total species richness of Oriental Diplopoda), the Paradoxosomatidae are largely epigean, in part because much of the collecting and taxonomic exploration efforts still focus on cavernicoles alone. Only the large (35 species, mainly epigean), basically Southeast Asian genus *Desmoxytes* Chamberlin, 1923, often referred to as “dragon millipedes”, encompasses 11 unquestioned troglobionts (e.g. Fig. 6), all confined to southern China (Golovatch, Geoffroy and Mauriès 2010a, Golovatch, Li et al. 2012, Liu et al. 2014). The small Indochinese genus *Piccola* Attems, 1953 is represented in southern China by one troglomorphic, likely troglobitic species (Liu and Tian, in preparation). Similarly, the rather small, Australasian family Haplodesmidae is largely represented by epigean species as well, however several presumed troglobionts (Figs 7, 8), especially from the largest genus *Eutrichodesmus* Silvestri, 1910 (with 45 described species), are known from Indochina and southern China (Golovatch et al. 2009b, 2009c, 2015, Golovatch, Mikhaljova et al. 2010, Liu and Tian 2013). The quite large, mainly tropical family Cryptodesmidae is also dominated by epigean species, but a few are presumed troglobionts both in the Neotropical and Oriental regions, including a species of *Trichopeltis* Pocock, 1894 in southern China (Golovatch et al. 2010).

In contrast, species of the small, generally Oriental family Opisotretidae (Polydesmida) also occur epigeically in the karst regions of southern China, but the few likely troglobionts have only been encountered in Sulawesi, Indonesia and in Papua New Guinea (Golovatch, Geoffroy et al. 2013). Furthermore, apparently none of the very few, partly still unidentified species of the family Trichopolydesmidae (Polydesmida), the order Glomeridesmida (both these groups pantropical), or the order Julida (basically Holarctic), which have occasionally been encountered in caves in Southeast Asia, seem to represent troglobionts.

Not only is the fauna of cavernicolous diplopods of China clearly biased to rather few lineages, but also the morphotypes they represent appear to be fewer compared to epigean counterparts. Thus, only juloid, glomeroid and polydesmoid morphotype millipedes occur among troglobionts in southern China.

The representation of millipede orders in the fauna of southern China, also roughly showing the proportion of presumed troglobionts, is summarized in Table 1.

## Conclusions

Generally speaking, southern China harbours a very rich and diverse fauna of Diplopoda, probably numbering several hundred species. It consists of not only clearly dominating Oriental elements, but also a proportion of Palaearctic ones. Yet only a highly limited number of lineages appear to have successfully colonized the cave environment, not only in China, but in the entire Oriental realm. Biogeographically, these lineages, however few, also demonstrate the dominance of presumably Oriental groups (Kashmireumatidae, Megalotylidae, Cambalopsidae, Sinocallipodidae, Paracortinidae, *Hyleoglomeris*, *Desmoxytes*, *Pacidesmus*, *Glenniea*, *Trichopeltis*, *Piccola*) over the clearly Palaearctic ones (*Bollmania*, *Epanerchodus*). In full agreement with common wisdom, the fauna is actually a mixture of components from both these realms, definitely with numerous further troglobitic species still to be revealed. Because truly cave-dwelling genera, tribes or families are nearly absent among the Chinese or even entire Oriental Diplopoda, future explorations seem far more likely to yield lots of further new species, but barely anything else of a higher taxonomic rank. The only remarkable exceptions in southern China are the endemic family Guizhousomatidae, monobasic, and the oligotypic genus *Lipseuma*, both these taxa likely highly relictual troglobionts. Continental China may well prove to support about 1,000 millipede species of various origins, mainly Oriental and Palaearctic. Most of this impressive diversity is expectedly confined to the warmer, highly montane, humid tropical and subtropical parts of the country where numerous karst massifs are also known to often dominate the landscapes.

The exceptional biotic richness and abounding local endemism of the karsts and their caves in southern China (Deharveng et al. 2008), where millipedes are among the dominant terrestrial groups showing particularly high rates of diversification (e.g. Deharveng and Bedos 2012), are certainly among the most vulnerable elements of biodiversity from a conservation point of view. Global change, coupled with an increasingly powerful anthropogenic pressure such as the removal or fragmentation of native woods often replaced by timber eucalypt or pine plantations (both inevitably followed by soil and water acidification), land development, woodworking, mining/cement and similarly detrimental industries, environmental pollution, water removal etc., must be regarded as the main targets and concerns of nature conservation in the region. Because we still know too little, it is highly difficult to impossible to provide meaningful estimates of the current threats faced by cave biodiversity in southern China, but the problems are certainly quite acute and the stakes pretty high. Not just millipedes, albeit one of the major components of cave and karst environments, but rather the karsts themselves deserve conservation, especially those where aboriginal woody and shrub vegetation still dominates, through a network of national or regional nature reserves and parks. This work is being planned or already underway, in Guangxi under the supervision and with the ongoing support of the Biodiversity Conservation Office, Department of Environmental Protection, Guangxi Regional Government, Nanning, China (e.g. Liu et al. 2014, Tian et al. 2014).
